# Retrobulbar Hemorrhage Secondary to Acquired Hemophilia A

**DOI:** 10.7759/cureus.17760

**Published:** 2021-09-06

**Authors:** Hishali D Jayasundara, Lasitha Y Herath, Keertie S Kularatne

**Affiliations:** 1 Medicine, National Hospital Kandy, Kandy, LKA

**Keywords:** cyclophosphamide, immunosuppression, inhibitors, retrobulbar hemorrhage, acquired hemophilia a

## Abstract

Acquired hemophilia A (AHA) is a different bleeding disorder to that of classic hemophilia A. It is an autoimmune-mediated bleeding disorder due to the presence of autoantibodies (inhibitors) directed against plasma coagulation factor VIII. This can result in fatal hemorrhage; thus, eradication of the antibody is the mainstay of treatment. Nontraumatic retrobulbar hemorrhage is a very rare presentation of hemophilia. We report an elderly male presenting with retrobulbar hemorrhage secondary to AHA who was successfully treated with immunosuppression.

## Introduction

Acquired hemophilia A is a rare bleeding disorder which is caused by autoantibodies directed against coagulation factor VIII (FVIII). Patients with no history of bleeding episodes in the absence of significant family history of bleeding disorders may present with spontaneous hemorrhage [1,2]. Characteristically, activated partial thromboplastin time (aPTT) will be prolonged with the rest of the clotting profile being normal. Majority of the patients are above 50 years. However, there are two peaks identified: a small peak between 20 and 30 years of age and a majority between 68 and 80 years [3,4]. This estimate is based on limited data since some patients with inhibitors against factor VIII may not be reported or would have not been diagnosed.

Fifty percent of the patients have no underlying disorder identified and therefore are known as the idiopathic form. Fifty percent have coexisting disorders such as autoimmune diseases as rheumatoid arthritis, Sjogren’s syndrome, inflammatory bowel disease, multiple sclerosis and other disease entities as diabetes mellitus, hepatitis, respiratory or dermatological diseases, hematological malignancies, solid tumors or secondary to drugs such as penicillin or interferon. Pregnancy-related cases are reported mainly in the postpartum period [1,2,5,6].

Patients are at risk of severe and fatal hemorrhage. Many can present with bleeding into the skin, muscles, soft tissues and mucous membranes and manifest as epistaxis, gastrointestinal, urological bleeds or retroperitoneal hematomas. Typical hemarthroses as in classic hemophilia A are unusual [4]. Ocular hemorrhage can be either traumatic or nontraumatic. However, nontraumatic ocular bleeding manifesting as a presentation of hemophilia is very uncommon [7]. Diagnosis and management of acquired hemophilia A (AHA) is complex. The main objective of treatment is to arrest bleeding, eradicate the autoantibodies directed against plasma coagulation factor VIII, treat any underlying condition and also protect the patient against trauma [2,8].

## Case presentation

A 64-year-old businessman presented to us with sudden onset of acute spontaneous bleeding into the right eye with loss of vision followed by blackish discoloration of the eye. Simultaneously, he developed multiple nontraumatic bruises over his upper and lower limbs along with melena. It was also associated with left hip pain and left-sided knee joint pain and swelling. He denied having gum bleeding, epistaxis, hemoptysis, hematuria or fresh per rectal bleeding. One month ago, he had sought medical advice due to melena where he was transfused with 3 pints of blood and discharged. He has had no significant childhood bleeding manifestations nor any significant family history of bleeding disorders or malignancies. He has had no associated fever, loss of appetite, weight loss, arthralgia, facial photosensitive rashes, oral ulcers, dry mouth or dry eyes nor any lumps. He denies any history of chronic cough, altered bowel habits nor any bladder outflow obstructive symptoms. He had not been on any antiplatelets or anticoagulants.

**Figure 1:**
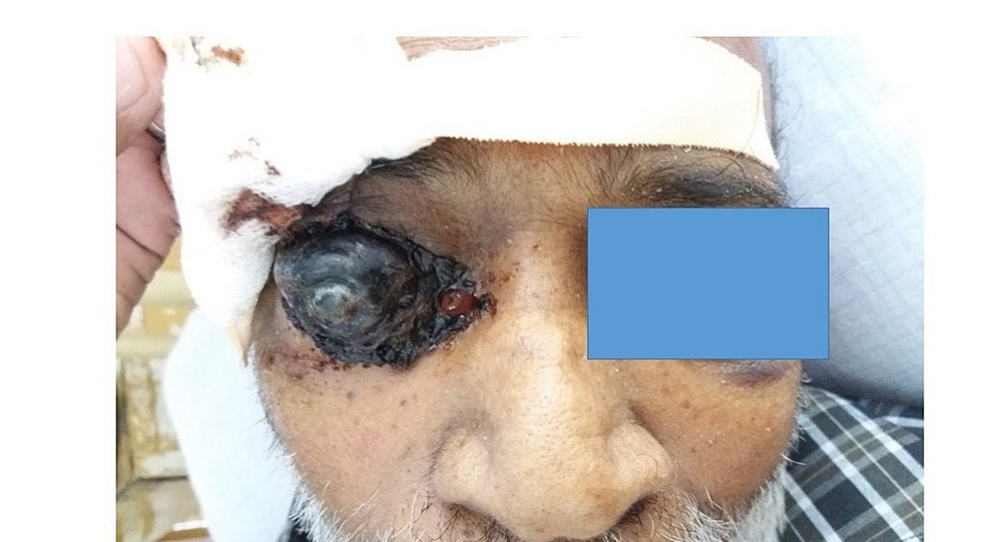
Gangrenous right-sided eye

**Figure 2:**
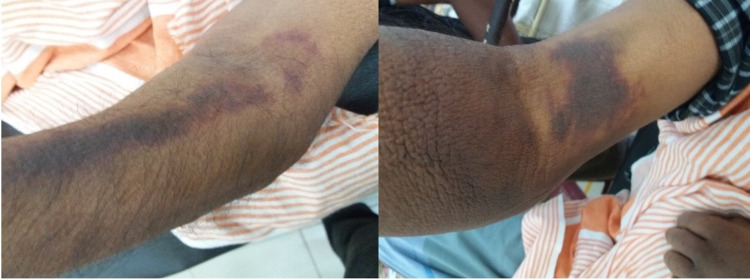
Multiple ecchymotic patches

Examination revealed a gangrenous right-sided eye as in Figure 1. He was mildly pale but not icteric. He had no lymphadenopathy but had grade IV clubbing with multiple ecchymotic patches mainly over both his upper limbs and the trunk as shown in Figure 2.

Left fundus revealed no hemorrhages, neither papilledema. He was hemodynamically stable with normal cardiovascular, respiratory and abdominal system examination findings. Left-sided straight leg raising test was positive, and there was mild swelling and tenderness over the left-sided knee joint. He had no features of any connective tissue disease. Digital rectal examination was also normal.

Investigations revealed an aPTT of 127sec (normal range: 25-35sec) and more than 120 repeatedly. Rest of the clotting profile was normal. Hemoglobin was 9.3g/dl (normal range: 13-17g/dl), and platelets were 588 x 109/L (normal range: 150-450 x 109/L). Coagulation mixing studies then revealed the presence of inhibitors against factor VIII. Facilities to detect factor levels via the Bethesda assay were not available in Kandy, and the patient was not in a condition to be taken to Colombo, which was approximately 100km away, due to the high risk of bleeding. Liver and kidneys functions were normal. The erythrocyte sedimentation rate was 70mm/hr, and C-reactive protein was normal. Noncontrast computed tomography of the brain was normal with no evidence of intracranial hemorrhage. After the confirmation of AHA, workup for a secondary cause was commenced. Peripheral blood smear revealed iron deficiency anemia with evidence of bleeding and showed no evidence of any hematological malignancy. Urgent bone marrow biopsy too did not reveal any abnormality. Chest X-ray was only hyperinflated, and mantoux was 6mm. Multiple myeloma screening which included a skeletal survey, urine for Bence-Jones proteins and serum protein electrophoresis were negative. Ultrasound scan of the abdomen and pelvis was normal except for a moderate left-sided psoas hematoma. Contrast-enhanced computed tomography of the chest and abdomen revealed no underlying malignancy except for the resolving psoas hematoma and changes in the lung parenchyma suggestive of healed pulmonary tuberculosis (TB) where the patient denies any significant history of TB. Tumor markers such as prostate-specific antigen, carcinoembryonic antigen and alpha-fetoprotein were also normal. Antinuclear antibodies and rheumatoid factor were not detected. Upper and lower gastrointestinal endoscopies did not reveal any malignant lesions.

Hematology opinion was sought on the day of admission; factor eight inhibitor bypass activity (FEIBA) was administered; and bleeding was arrested. Intravenous tranexamic acid along with intravenous omeprazole infusion was administered for two days until melena settled. He was transfused with blood on the following day. Ophthalmology opinion was taken with regards to enucleation, but the procedure was not encouraged until the patient’s aPTT was normalized due to the very high risk of postoperative bleeding. High-dose prednisolone of 60mg was commenced on the fifth day following admission. His blood sugars were monitored daily. Simultaneously, azathioprine 100mg was commenced and then increased to 150mg daily dose.

His bleeding was arrested, and with time, the aPTT dropped to 80sec (normal range: 25-35sec) but only after about a month. Since his target aPTT was not achieved even after a month of azathioprine treatment, it was omitted and substituted with intravenous cyclophosphamide. Cyclophosphamide was continued for two weeks along with adequate hydration while anticipating for any of its side effects. Prednisolone was continued and gradually tapered off. He responded well; his aPTT normalized very rapidly by two weeks; and he did not develop any complications of cyclophosphamide. There was spontaneous enucleation of the eyeball during the hospital stay. No surgical intervention was suggested by the ophthalmology team even after the normalization of aPTT. He was prophylactically administered the meningitic dose (2g twice a day) of ceftriaxone for 14 days so as to prevent any complications. Cyclophosphamide was continued for a duration of six weeks.

He was discharged after six weeks of hospital stay and reviewed monthly with an aPTT. Prednisolone dose was gradually tapered and omitted. He was then advised to come back for a yearly physical review and an aPTT.

## Discussion

Although acquired hemophilia A is a rare bleeding disorder, it is a potentially life-threatening bleeding disorder. Its incidence increases with age and is an uncommon disease among children. Early diagnosis and prompt treatment is essential to prevent fatal hemorrhage. Immunosuppression is the mainstay of treatment. There is a diversity in the use of these immunosuppressive drugs, and almost all documented here had been successful according to literature review. For therapeutic strategies, quantitative assay of factor VIII inhibitor is essential. The Bethesda method is the most often performed method. A low inhibitor titer is defined as less than 10 BU, intermediate as 10 to 20 BU, and high as more than 20 BU [9]. Prednisolone treatment alone may be associated with complete remission in 32% of patients, and addition of cyclophosphamide increases response to 70% [10].

Patient’s age is an important factor in determining the outcome. Long-term immunosuppression predisposes patients to infection, thus increasing the mortality; therefore, rapid elimination of the inhibitor is important. A therapeutic approach for AHA called the modified Bonn-Malmö protocol (MBMP) was developed in view of suppressing bleeding, eliminating inhibitors permanently and developing immune tolerance. The protocol included immunoadsorption for inhibitor elimination, factor VIII substitution, intravenous immunoglobulin and immunosuppression. Thus, the high levels of autoantibodies and immune complexes are removed from the patient's plasma via this large-volume immunoadsorption [11].

In our setup, the first recommendation was FEIBA. This was only a temporary resolution and was not freely available, very expensive and of limited stock. This prompted us to search for a more efficacious and a targeted therapy. Some centers have documentation of successful treatment of AHA with immunosuppression using continuous low-dose oral cyclophosphamide and prednisone. They had observed progressive decline in titers of factor VIII inhibitor with continued therapy and the resolution of recurrences of inhibitors with retreatment [12]. We too practiced this method, and it was successful. The only method of assessing the response was by doing aPTT along with clinical assessment looking for bleeding since we had no facilities to do inhibitor levels. Studies have also shown that administering steroids combined with high-dose immunoglobulin and desmopressin was well tolerated and effective in treating elderly patients with high titers of factor VIII inhibitors [13]. Desmopressin has a role in the treatment of non-life-threatening hemorrhage in patients with AHA having low titers of inhibitors (<5 Bethesda units or factor VIII ≧5%) [4]. Another center had used rituximab as the first treatment modality. They had noted that the time taken for the inhibitors to resolve ranged between 3 and 12 weeks, and patients showed a quick response on the day after administration in comparison to the conjunction of prednisolone and cyclophosphamide which took about 3 to 37 weeks for the inhibitors to resolve [6]. Management of AHA during pregnancy is similar to nonpregnant individuals. The most relevant aspect of management is prevention and/or control of bleeding. Immunosuppression with prednisolone may be used but not cyclophosphamide. The primary goal of treatment for an acquired factor VIII inhibitor is cessation of bleeding, followed ultimately by a decrease in the titer of the inhibitor. The former is monitored via the usual clinical and laboratory observations (e.g., observable blood loss, blood in urine or stool, repeated blood counts). Since the titers of inhibitors drop very slowly following successful treatment, it is neither necessary nor advisable to check the patient’s aPTT or inhibitor titer more often than every two to four weeks once immunosuppressive therapy has been started. In our patient, weekly review of aPTT for about a month was static. He was then reviewed monthly for six months and then annually. About 20% tend to relapse after achieving complete remission, and 70% of such relapsing patients achieve a second complete remission [14].

After extensive investigation for a secondary cause for acquired hemophilia A, we could not identify any. As mentioned, 50% will be idiopathic. We highlight a rare and life-threatening complication of acquired hemophilia A where our patient presented with retrobulbar hemorrhage and complete vision loss.

## Conclusions

Retrobulbar hemorrhage secondary to acquired hemophilia A is a rare clinical presentation, and there may be a delay in diagnosing this. Clinical suspicion and simultaneous vigorous symptomatic treatment are very important to arrest bleeding, followed by immunosuppression as early as possible to eradicate the inhibitors.
